# Liquid–Liquid and Liquid–Solid Interfacial Nanoarchitectonics

**DOI:** 10.3390/molecules29133168

**Published:** 2024-07-03

**Authors:** Katsuhiko Ariga

**Affiliations:** 1 Research Center for Materials Nanoarchitectonics (MANA), National Institute for Materials Science (NIMS), Ibaraki 305-0044, Japan; ariga.katsuhiko@nims.go.jp; 2 Graduate School of Frontier Sciences, The University of Tokyo, Chiba 277-8561, Japan

**Keywords:** covalent organic framework, liquid–liquid interface, liquid–solid interface, living cell, metal-organic framework, molecular assembly, nanoarchitectonics, organic semiconductor

## Abstract

Nanoscale science is becoming increasingly important and prominent, and further development will necessitate integration with other material chemistries. In other words, it involves the construction of a methodology to build up materials based on nanoscale knowledge. This is also the beginning of the concept of post-nanotechnology. This role belongs to nanoarchitectonics, which has been rapidly developing in recent years. However, the scope of application of nanoarchitectonics is wide, and it is somewhat difficult to compile everything. Therefore, this review article will introduce the concepts of liquid and interface, which are the keywords for the organization of functional material systems in biological systems. The target interfaces are liquid–liquid interface, liquid–solid interface, and so on. Recent examples are summarized under the categories of molecular assembly, metal-organic framework and covalent organic framework, and living cell. In addition, the latest research on the liquid interfacial nanoarchitectonics of organic semiconductor film is also discussed. The final conclusive section summarizes these features and discusses the necessary components for the development of liquid interfacial nanoarchitectonics.

## 1. Introduction

The world moves quickly, and various problems appear successively. Science and technology must keep up in finding solutions to these problems. Research on energy [1,2,3,4,5,6,7,8,9,10], environmental [11,12,13,14,15,16,17,18,19,20], and biomedical problems [21,22,23,24,25,26,27,28,29,30] is being conducted, spanning basic and applied perspectives. In addition, basic research and the development of devices that contribute to information technology are being conducted to build a new society [31,32,33,34,35,36,37,38,39,40]. To address these issues, it is essential to develop precisely organized functional materials. Mankind is tackling this problem tirelessly. Yet, the mechanisms to deal with these problems already exist in biological systems. Biological systems are very efficient and highly selective. Moreover, they operate under mild conditions and normal temperature and pressure. The secret is that in a biofunctional system, functional components are very intricately organized [41,42,43,44]. They work in tandem to achieve high functionality. A pre-existing methodology is there for reference. Living systems have developed such excellent systems over billions of years of evolution, and even though their organizational structures are highly diverse, there are a few common features. One is that they are constructed in liquid systems (aqueous solution systems). In living organisms, all functions occur in aqueous environments. Another feature is the use of interfaces. Biological mechanisms rarely take place in free solution. They always work at interfaces, such as cell membrane surfaces, the internal surfaces of protein pockets, and surfaces of biopolymers such as DNA. In other words, the key to the organization and expression of high functions in biological systems is twofold: the fact that they take place in liquid and in interfacial environments. The need for materials design to take advantage of these characteristics in artificial systems will be reiterated later in this introductory section. Liquid–liquid interfaces and liquid–solid interfaces are the key.

In comparison to the development of functional structures in living systems, which has developed over billions of years, humans have developed methodologies for creating functional materials in a much shorter time. Let us take a brief look at the development of materials by mankind. The development of human society depends on the materials that become available and the tools made from them. It was not until the 20th century that the methods of material creation were systematized as a discipline. The 20th century saw the systematization of the methods of substance creation as a discipline, the inception and development of various types of chemistry. In particular, the development of material chemistry owes much to the development of chemistry, which involves making various things from molecules. In addition, the development of physics to evaluate the fabricated materials was also essential. Furthermore, the development of biology, which deepens our relationship with nature, is also important to utilize science and technology for our daily life and health. Among these, chemistry, which makes materials, continues to develop new functional materials even today. Organic chemistry [45,46,47,48,49,50,51,52,53,54], inorganic chemistry [55,56,57,58,59,60,61,62,63,64], polymer chemistry [65,66,67,68,69,70,71,72,73,74], supramolecular chemistry [75,76,77,78,79,80,81,82,83,84], co-ordination chemistry [85,86,87,88,89,90,91,92,93,94], biochemistry [95,96,97,98,99,100,101,102,103,104], and other material chemistry [105,106,107,108,109,110,111,112,113,114] are still creating various new substances, systems, and principles. At the same time, physical chemistry [115,116,117,118], analytical chemistry [119,120,121,122,123], and interfacial chemistry [124,125,126,127,128] also continue to develop, in addition to physical methods for material properties [129,130,131,132]. Throughout this historical development, there is one fact that physics and chemistry have elucidated. It is the principle that properties and functions depend not only on the nature of the substance itself, but also on its external and internal structures [133,134,135]. In other words, control of structure and organization is essential for better functionality. In particular, control of the structure at the nanoscale level is crucial.

The founding of nanotechnology was pivotal in this research trend. Nanotechnology plays a central role in modern chemistry and is involved in various sciences and technologies. The observation of structure and motion at the atomic, molecular, and nanolevel [136,137,138,139], as well as the elucidation of specific physical properties in such small areas [140,141,142,143], are being studied. As these studies progress, the importance of nanoscale science is becoming increasingly prominent. Once the importance of nanoscale phenomena is well understood, it must be integrated with other material chemistries. This is the integration of understanding at the nanoscale with the technology of materials science. In other words, it is the construction of a methodology to build up materials based on nanoscale knowledge. This is also the beginning of the concept of post-nanotechnology [144]. This role is played by the concept of nanoarchitectonics, which has been rapidly developing in recent years. Just as Richard Feynman founded nanotechnology in the middle of the 20th century [145,146], nanoarchitectonics was proposed by Masakazu Aono at the beginning of the 21st century [147,148].

**Figure 1:**
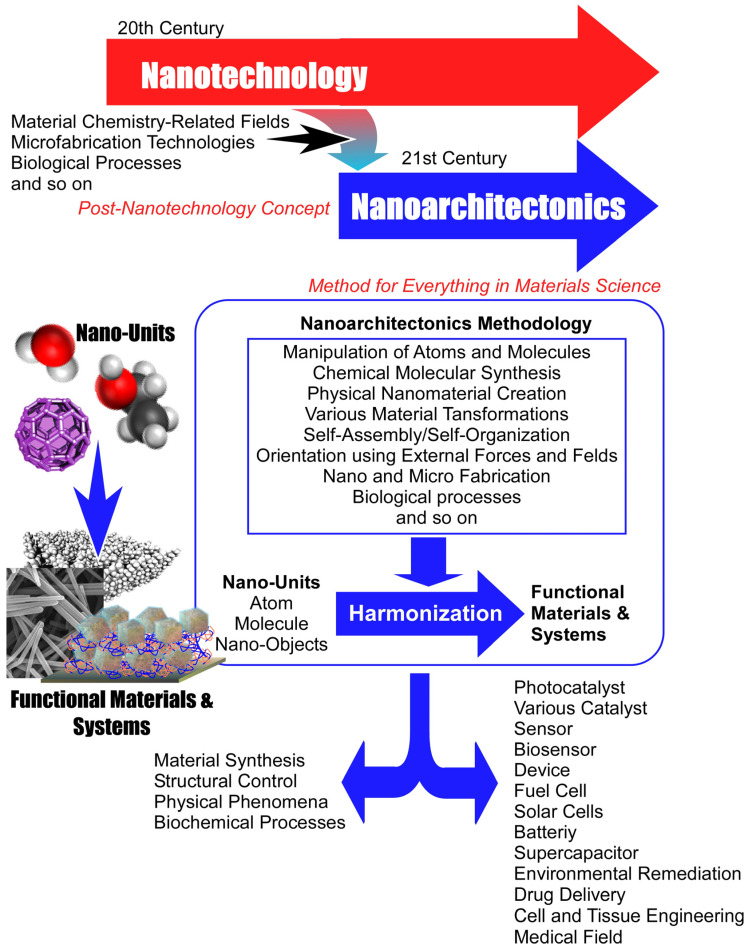
Outline, history, and effects of the nanoarchitectonics concept.

Nanoarchitectonics is a methodology for creating functional material systems by assembling nanounits such as atoms, molecules, and nano-objects (Figure 1) [149,150]. It can be thought of as architecture in the nano or material world. Methods of creating materials by assembling molecules and other materials have been also studied in supramolecular chemistry and other fields. Supramolecular assemblies have been formed through intermolecular interactions [151,152,153,154,155], inorganic mesoporous materials have been created through template synthesis [156,157,158,159,160], metal-organic frame work (MOF) with precise porous structures have been created through co-ordination chemistry [161,162,163,164,165], similar porous materials covalent organic framework (COF) can be constructed by polymer chemistry [166,167,168,169,170], and self-assembled monolayer (SAM) [171,172,173,174,175], Langmuir–Blodgett (LB) method [176,177,178,179,180], and layer-by-layer (LbL) assembly [181,182,183,184,185] have been developed with interface science. Although they have been successful, they are not unified and are rather independent. Nanoarchitectonics integrates these technologies into a unified field. Nanoarchitectonics is the broad integration of nanotechnology, including these material chemistry-related fields, various microfabrication technologies, biological processes, and so on. It must be emphasized that rather than the creation of a new field, nanoarchitectonics is more of an integration of fields and a proposal for a unified concept.

Therefore, material architecture through nanoarchitectonics will include a variety of elemental technologies: manipulation of atoms and molecules, chemical molecular synthesis, physical nanomaterial creation, various material transformations, self-assembly/self-organization, orientation using external forces and fields, nano and micro fabrication, biological processes, etc., in selected combinations, architecture of matter [186]. With these processes, materials construction often becomes multistep. Therefore, one of the characteristics of nanoarchitectonics is that it is easier to create hierarchical and asymmetric structures [187] than by simple self-assembly and other systems consisting of a single equilibrium [188]. In addition, phenomena in the nanoscale region are easily affected by various perturbations. For example, the effects of thermal fluctuations, stochastic distributions, and quantum effects might be involved. The approach of nanoarchitectonics is to harmonize the effects while balancing those ambiguities [189]. Hierarchical structures and the harmonization of several effects are similar to the characteristics of the organization of functional structures in biological systems. Nanoarchitectonics has the propensity to architect functional structures similar to those in biological systems [190,191]. In addition, the ultimate goal of nanoarchitectonics would be to create highly functional structures like those of living systems.

The above principles of nanoarchitectonics are very general. The target materials and their applications are not limited. The scientific papers advocating the term “nanoarchitectonics” cover a wide range of applications. It is used in basic areas such as material synthesis [192,193,194,195,196,197,198], structural control [199,200,201,202,203,204,205], the pursuit of physical phenomena [206,207,208,209,210,211,212], and basic biochemical processes [213,214,215,216,217,218,219]. Nanoarchitectonics is also widely applied in photocatalysts [220,221,222,223,224,225,226], various catalysts [227,228,229,230,231,232,233], sensors [234,235,236,237,238,239,240], biosensors [241,242,243,244,245,246,247], devices [248,249,250,251,252,253,254], fuel cells [255,256,257,258,259,260,261], solar cells [262,263,264,265,266,267,268], various batteries [269,270,271,272,273,274,275], supercapacitors [276,277,278,279,280,281,282], other energy systems [283,284,285,286,287,288,289], environmental remediation [290,291,292,293,294,295,296], drug delivery [297,298,299,300,301,302,303], cell and tissue engineering [304,305,306,307,308,309,310], and in medical fields [311,312,313,314,315,316,317]. Nanoarchitectonics is the concept of building functional material systems from atomic and molecular units. Because matter is originally composed of atoms and molecules, nanoarchitectonics can be a concept related to the creation of all matter. In physics, the ultimate physical principle is called “the theory of everything” [318]. Similarly, nanoarchitectonics can be a method for everything in materials science [319,320]. In fact, various materials development research projects that have not advocated nanoarchitectonics can be considered as nanoarchitectonics approaches under a broad concept.

Thus, the development of material science has culminated in the concept of nanoarchitectonics. However, the scope of application of nanoarchitectonics is wide, and it is somewhat difficult to encompass everything. Therefore, this review article will introduce the concepts of liquid and interface, which are keywords in the organization of functional material systems in biological systems, as mentioned above. At the interface, the movement and location of materials are restricted. In addition, phenomena unique to the interface also occur [321,322]. The interface is an attractive medium to develop nanoarchitectonics [323,324]. From this perspective, nanoarchitectonics for the LB method [325,326,327] and LbL assembly [328,329,330,331], which are interfacial processes, have been reviewed so far. In contrast, this review paper discusses nanoarchitectonics for interfaces in the liquid phase. The target interfaces are liquid–liquid interface, liquid–solid interface, and so on. As there are so many different examples falling under these fields, it is not necessarily appropriate to take an all-encompassing approach. A methodology that focuses on a few recent examples and extracts their characteristics is more practical. In this review paper, such cases are summarized under the categories of molecular assembly, MOF and COF, and living cell. In addition, the latest research on the liquid interfacial nanoarchitectonics of organic semiconductor film is also discussed. The final conclusive section summarizes these features and discusses what is needed for the development of liquid interfacial nanoarchitectonics and nanoarchitectonics in general.

## 2. Molecular Assembly

A liquid–liquid interface is a powerful site for the creation of nanostructures and materials from molecules. One such method is the liquid–liquid interfacial precipitation method [332]. This is a method to create structures by inducing molecular assembly at the interface created by immiscible solvents. The target molecule is dissolved in a good solvent, and a poor solvent is added to create a liquid–liquid interface. Then, due to the difference in solubility between the two solvents, insoluble aggregates or crystals of the target molecules are formed at the liquid–liquid interface. Depending on parameters such as the combination of solvents and the concentration of molecules in the good solvent, the structure and shape of the precipitated object will change. This is one method of nanoarchitectonics of materials from molecules. An interesting research example is the nanoarchitectonics of fullerenes such as C_60_ and C_70_ at the liquid–liquid interface. Fullerenes are composed of only one element, carbon, and are essentially spherical in shape. They have no functional groups with which to interact. It is a simple entity: a single element and zero dimensionality. When such fullerenes are subjected to liquid–liquid interfacial precipitation, a wide variety of structures are obtained. This is a remarkable example of the diversity of nanoarchitectonics. One-dimensional nanowhiskers [333], nanorods [334], nanotubes [335], two-dimensional nanosheets [336,337], and three-dimensional nanocubes [338] are obtained by the liquid–liquid interfacial precipitation of fullerenes. These can be controlled simply by changing the combination of solvents, fullerene concentration, and mixing conditions. The diversity of structures can be further increased by coexisting or reacting compounds, or by post-treatment with solvents or different reagents. Complex and hierarchical structures can be obtained, such as rods extending from a cube [339], holes on each face of a cube [340], and holes in a cube like a gyrode [341], as well as structural changes analogous to biological differentiation and metamorphosis, such as the gradual growth of a tail from a spherical assembly [342]. From simple structures such as fullerenes, liquid–liquid interfacial nanoarchitectonics has the potential to produce a wide variety of structures with extremely simple manipulations.

**Figure 2:**
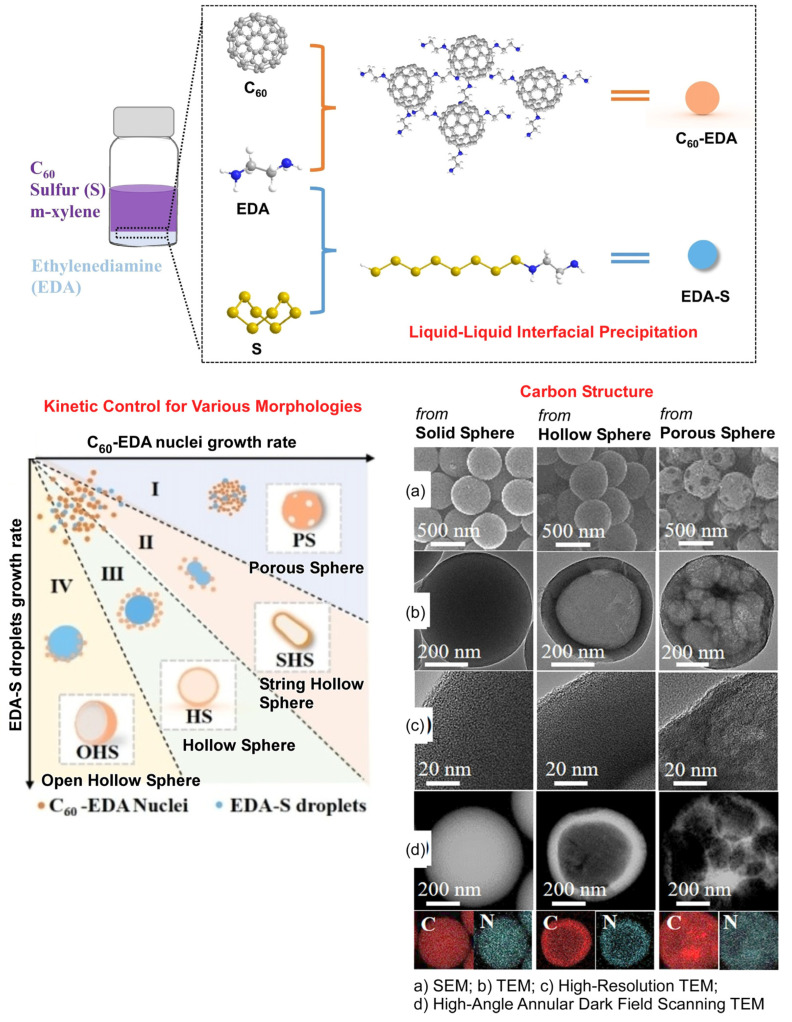
Kinetically controlled liquid–liquid interfacial precipitation (KC-LLIP) method to produce porous spheres, string hollow spheres, hollow spheres, and open hollow spheres from C_60_ with the aid of ethylenediamine and sulfur. The liquid–liquid interfacial precipitation method, kinetic control for various morphologies, and resulting carbon structures are explained. Reprinted with permission from [343]. Copyright 2022 Wiley-VCH.

Although the liquid–liquid interfacial precipitation strategy described above can be diversified by combining various components, the kinetic control process can further lead to a variety of morphological controls. Pursuant to this strategy, Chen et al. have successfully synthesized C_60_ spheres and their carbon materials of various sizes and morphologies by using droplets as templates (Figure 2) [343]. This method would allow for the mass production of solid or hollow C_60_ nanospheres with controlled sizes. The form in which the nanospheres are nanoarchitectonized is a matter of kinetically controlling the liquid–liquid interfacial precipitation process. This can be thought of as the kinetically controlled liquid–liquid interfacial precipitation (KC-LLIP) method. Specifically, 1,2-ethylenediamine was used as a covalent crosslinker for C_60_ molecules. It contributes to the in situ generation of ethylenediamine-C_60_ shells. The formation of ethylenediamine-C_60_ product was controlled by the addition of isopropyl alcohol. Sulfur was then added and the ethylenediamine-sulfur droplets were used as egg yolks. These droplets are important in creating a hollow structure via the formation of the yolk-shell structure. The nucleus of the 1,2-ethylenediamine-C_60_ complex adsorbs on the surface of the ethylenediamine-sulfur droplets. The former nuclei grow to form a shell of the ethylenediamine-C_60_ complex and the 1,2-ethylenediamine-sulfur droplets are removed by washing. As a result, hollow structures are obtained. The diversity of the C_60_ sphere structures produced is due to the regulation of the rate of structural growth of ethylenediamine-sulfur droplets and ethylenediamine-C_60_ complexes. Accordingly, the KC-LLIP method yields various forms of fullerene aggregates. The formations of porous spheres, string hollow spheres, hollow spheres, and open hollow spheres were observed. Because the morphology and dimensions of these C_60_ spheres can be tuned, they are expected to have a variety of applications. For example, they can be used as novel catalysts and catalyst supports. For this purpose, it is necessary to convert C_60_ aggregates into carbon materials using calcination treatment. Solid spheres, hollow spheres, and porous spheres were calcinized at 750 ℃. Basically, they were carbonized while retaining their shape. High-resolution TEM suggested an amorphous carbon structure. Elemental mapping indicated a homogeneous distribution of carbon and nitrogen in these carbonized structures. The nitrogen-doped carbon sphere has an increased catalytic reaction surface due to the cavity structure. It is also a promising material as a catalyst and catalyst support because it prevents leakage of catalytic components. The exploration of such carbon nanomaterial synthesis strategies is crucial for the development of novel materials, as carbon spheres have a wide variety of potential applications in adsorbents, drug delivery, energy conversion and storage, and nanodevices, in addition to catalytic applications. The liquid–liquid interfacial nanoarchitectonics of kinetic control presented above could be a novel and groundbreaking approach. This simple process can yield carbon nanomaterials in a variety of forms, including size-defined solid spheres and hollow spheres with controllable pores and cavities. Based on this tactic, the advancement of nanoarchitectonics for the effective development of carbon materials for advanced applications is expected.

**Figure 3:**
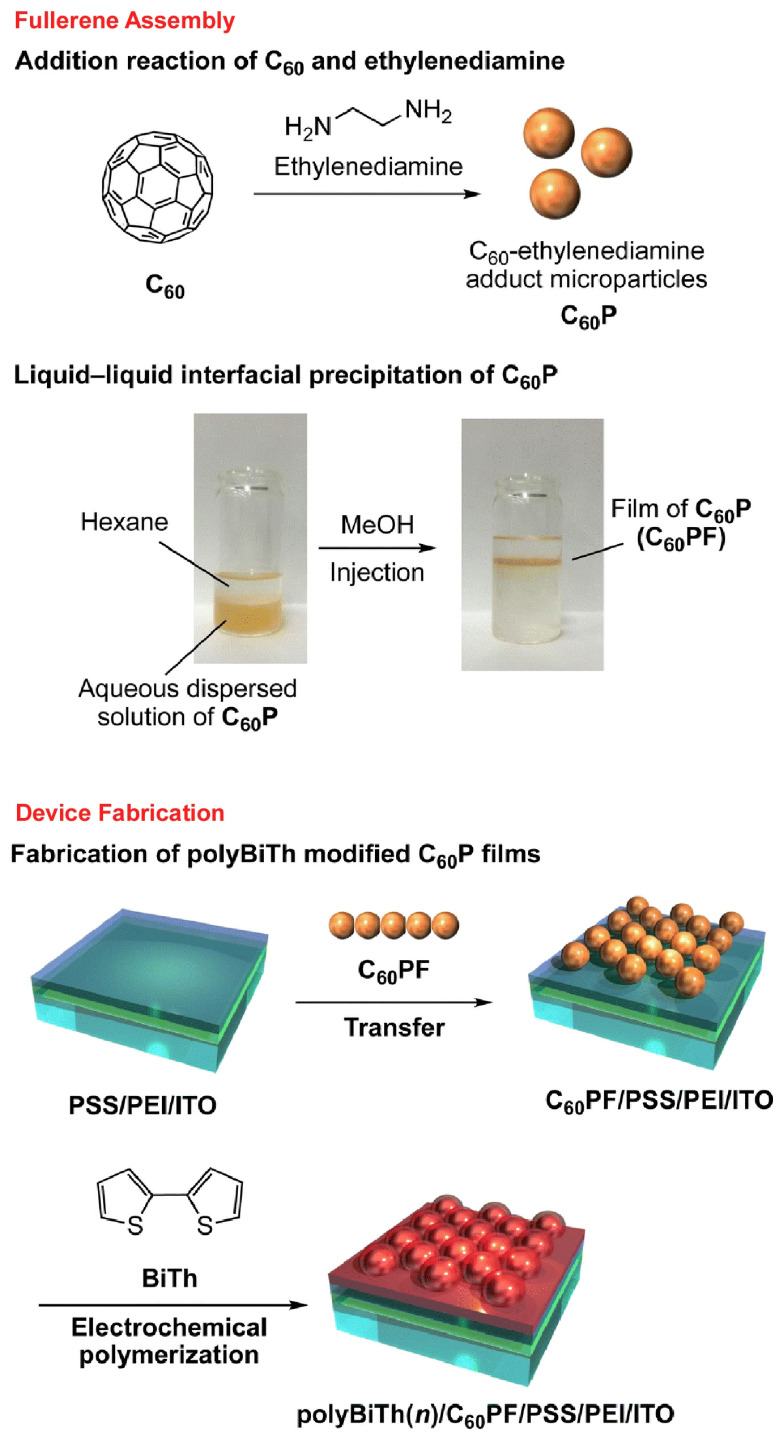
Preparation of thin films of ethylenediamine-modified C_60_ nanoparticles on an ITO electrode modified with alternating adsorbed layers of polyelectrolyte for a device with their unique photochemical and electrochemical properties. Fullerene assembly (**top**) and device preparation (**bottom**) are explained. Reproduced under terms of the CC-BY license [344]. Copyright 2023 Royal Society of Chemistry.

Several studies have examined device properties of fullerene assemblies prepared by liquid–liquid interfacial nanoarchitectonics. Akiyama and co-workers prepared thin films of ethylenediamine-modified C_60_ nanoparticles and investigated their unique photochemical and electrochemical properties (Figure 3) [344]. The fluorescence and photocurrent generation properties of ITO electrodes modified with composite films with polythiophene were investigated. Ethylenediamine-C_60_ composite particles were prepared using liquid–liquid interfacial precipitation. A thin film of ethylenediamine-C_60_ composite microparticles was coated onto an ITO electrode modified with alternating adsorbed layers of polyelectrolyte. In addition, polythiophene-modified, densely packed ethylenediamine-C_60_ particulate films were prepared using a combination of electrochemical polymerization of 2,2′-bithiophene. The fluorescent emission properties of the nanoarchitectonized composite films suggest that the ethylenediamine-C_60_-adduct particulate film functions not only as an electron acceptor for polythiophene but also as a photosensitizer. The partial photocellular properties of electrodes composed of ethylenediamine-C_60_-adduct particulate films and polythiophene were investigated. In the presence of methyl viologen, cathodic photocurrent was generated by excitation of the composite films. The obtained results suggest various possibilities of composite thin films of ethylenediamine-C_60_-adduct particulate films and polythiophene. In particular, the possibility of application to photoelectrochemical devices such as photoelectric conversion devices and sensors is promising.

Because liquid–liquid interfacial nanoarchitectonics is a powerful tool for structuring functional molecules other than fullerenes, Takase, Shimizu, and co-workers have prepared a highly conductive charge-transfer complex, (phthalocyaninato)cobalt iodide, at the liquid interface [345]. It is very important to make cobalt phthalocyanine industrially practical as a catalyst for CO_2_ reduction. For this purpose, it is necessary to study the influence of the conductivity enhancement of cobalt phthalocyanine crystals on the catalytic activity. From this perspective, a highly conductive charge-transfer complex, (phthalocyaninato)cobalt iodide, was investigated. In this approach, (phthalocyaninato)cobalt iodide is easily assembled by simply mixing a KI solution containing trifluoroacetic acid and cobalt phthalocyanine with a CH_2_Cl_2_ solution at the interface, applying the cobalt phthalocyanine crystal phase transition method. Moreover, UV–Vis analysis showed that I^−^ is changed to I_3_^−^ at the interface. The resulting material consists of a one-dimensional column of cobalt phthalocyanine and a linear array of I_3_^−^. The catalytic properties of (phthalocyaninato)cobalt iodide were investigated using polarization measurements and electrochemical impedance spectroscopy using a gas diffusion carbon electrode. The results showed high catalytic activity for CO_2_ reduction. In addition, high CO formation selectivity was obtained. This liquid–liquid interfacial nanoarchitectonics method has the potential to yield a variety of charge transfer complexes with I_3_^−^. Such nanoarchitectonized materials are expected to be applied to electrochemical devices.

**Figure 4:**
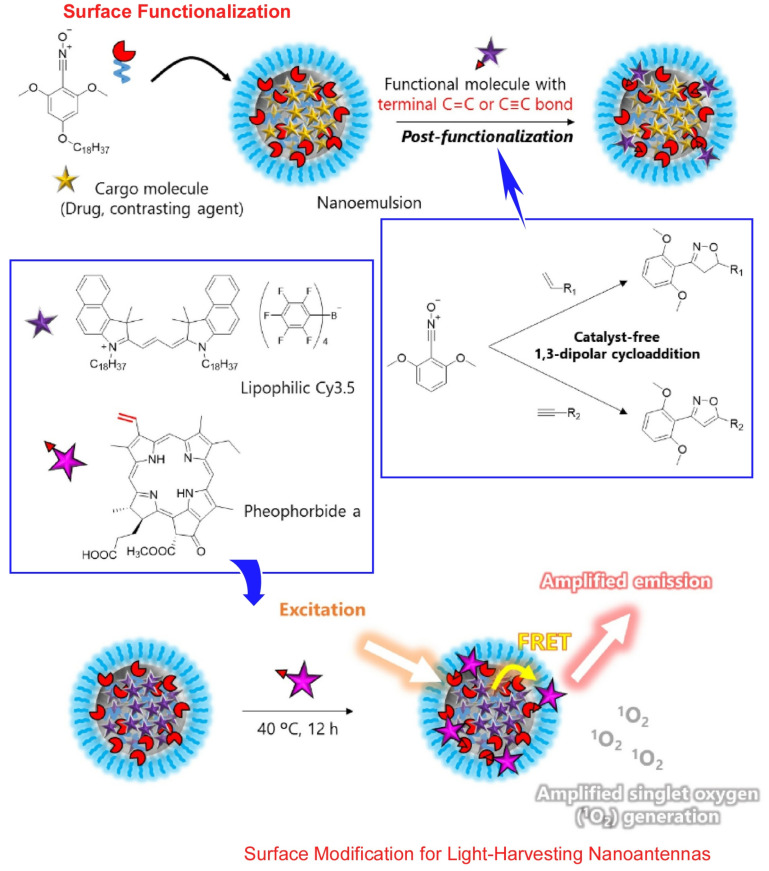
Introduction of lipophilic nitrile N-oxide compound for surface-functionalized nanoemulsions containing lipophilic cyanine 3.5 and a fluorescent dye molecule, where further functionalization with pheophorbide a, as a photosensitizer, generates singlet oxygen for photodynamic therapy. Surface functionalization (**top**) and surface modification for light-harvesting nanoantennae (**bottom**) are explained. Reprinted with permission from [346]. Copyright 2023 Oxford University Press.

Nanoemulsion provides a microscopic liquid–liquid interface. This environment is also an excellent environment in which to develop molecular nanoarchitectonics. However, for therapeutic and diagnostic applications of nanoemulsions, it is necessary to introduce appropriate functional groups at the interface. In particular, it is essential to develop methods for modifying liquid–liquid (oil–water) interfaces stabilized by surfactants. Modification techniques using lipophilic nitrile N-oxide compounds were developed by Niko and co-workers (Figure 4) [346]. The lipophilic nitrile N-oxide compounds are highly soluble in oil. Therefore, they can be easily introduced into the interface of nanoemulsions. The lipophilic nitrile N-oxide compounds introduced at the oil–water interface reacted efficiently with functional molecules having C=C or C≡C bonds at the ends under catalyst-free conditions. This is a 1,3-dipolar cycloaddition reaction. Surface-functionalized nanoemulsions can be used without any distinctive purification. Nanoemulsions containing lipophilic cyanine 3.5, a fluorescent dye molecule, and lipophilic nitrile N-oxide compounds were used for nanoarchitectonics. They were further functionalized with pheophorbide a, a photosensitizer that generates singlet oxygen for photodynamic therapy. This modification was confirmed by Förster resonance energy transfer analysis. When the lipophilic cyanine 3.5 molecules in the nanoemulsions were photoexcited, the excitation energy was efficiently transferred to pheophorbide a via Förster resonance energy transfer. These nanoemulsions can function as light-focusing nanoantennae. Therefore, this nanoemulsion system showed 7–18 times more efficient singlet oxygen generation than direct excitation of pheophorbide a. It was confirmed that despite the reduced cell permeability, efficient singlet oxygen generation induces cancer cell death. Surface modification techniques based on lipophilic nitrile N-oxide compounds are expected to be both practically useful and promising. The development of theragnostic materials based on nanoemulsions for selective tumor targeting, for example, is expected. Furthermore, this interfacial nanoarchitectonics approach could be applied to surface modification of other types of nanomaterials, such as polymeric nanoassemblies, micelles, and liposomes. In turn, it can be a promising method for imparting various functional groups and functions to bionanomaterials.

## 3. MOF and COF

This section focuses on metal-organic frameworks (MOFs) [347,348,349,350,351] and covalent-organic frameworks (COFs) [352,353,354,355,356] as targets for liquid interfacial nanoarchitectonics. Nanoporous materials have been actively studied from the viewpoint of various applications, and MOFs and COFs are representative categories. MOFs build up regular porous structures through co-ordination chemistry, whereas COFs do so through polymer chemistry. Both approaches are typical of nanoarchitectonics, which builds functional structures from building blocks. They are often prepared as two-dimensional structures at liquid interfaces [357,358,359]. MOF and COF can be representative players in liquid interfacial nanoarchitectonics.

In particular, these structures hold much promise from an application standpoint. MOFs have a large surface area and can store a large amount of photogenerated charge on their surface sites. Applications in photoelectrochemical capacitors are being considered. Photoelectrochemical capacitors have attracted attention for their charge accumulation and dissipation mechanisms, as well as for their spike and overshoot current properties. These properties are of interest for biomedical applications such as optical stimulation of nerves and sensing of biomolecules, etc. Moribe et al. have conducted a fundamental exploration into the application of MOFs to photoelectrochemical capacitors [360]. Transient photocurrent measurements were performed in a photoelectrochemical capacitor cell consisting of a porphyrin zirconium MOF electrode at the liquid–solid interface of phosphate-buffered saline and an electrode. In transient photoelectrochemical capacitor cell transient photocurrent measurements using MOF electrodes in phosphate buffer solution under an argon atmosphere, spike and overshoot photocurrents were observed. A clear growth and decay of cathodic current was observed during light irradiation. When the light was turned off, an anodic reverse current was generated, inducing spike and overshoot currents. The direction of the induced current indicated that the MOF electrode behaved like a p-type photoelectrochemical capacitor cell electrode. No spike or overshoot currents were observed when excess oxygen was introduced into the electrolyte. The obtained results suggest that the porphyrin zirconium MOF electrode accumulates charge at the surface site in the MOF pore closest to the electrolyte–electrode interface, which is the liquid–solid interface. Thus, porphyrin zirconium MOFs are expected to be promising for biomedical applications as photoelectrochemical capacitors. This is because quantitative sensing of biological phenomena, such as those seen in the photostimulation of neurons, may be possible. This has potential for future biomedical applications. Investigation and improvement of the stability of the MOF electrode in the biological environment is also very important for its practical application.

**Figure 5:**
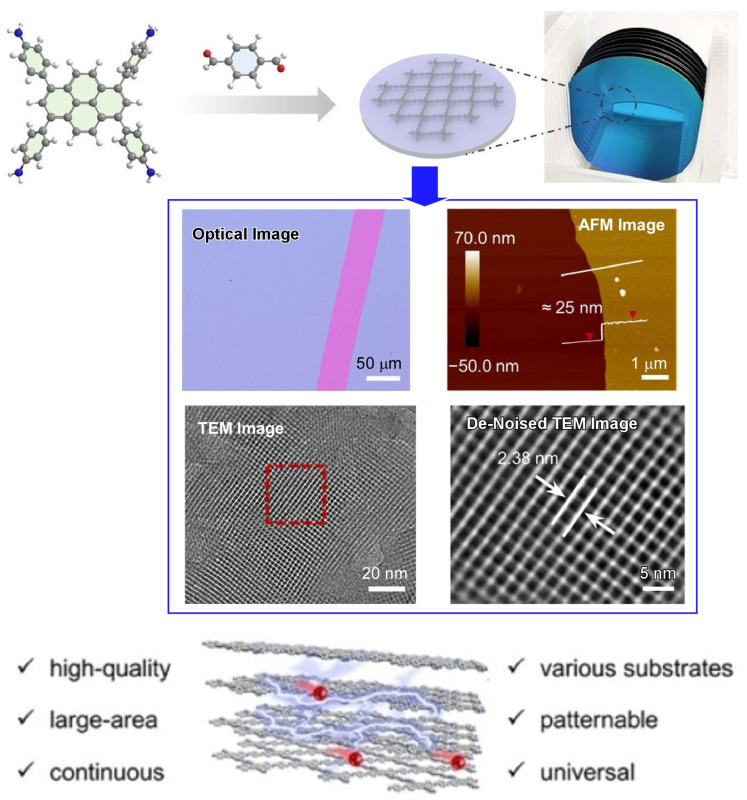
Preparation of COF films by polymerization at the liquid–solid interface under simple and mild conditions to result in various good features of a large lateral size, controllable thickness, high crystallinity, uniformity, and being free of contamination, wrinkles, and damage. Fabrication method (**top**), images (**middle**), and typical features (**bottom**) are explained. Reprinted with permission from [361]. Copyright 2024 Wiley-VCH.

COF is also used for a variety of applications, including various devices. For example, the construction of uniform COF films on electronic device substrates is a desirable technique to be established. Chen and co-workers have developed a method to prepare COF films by polymerization at the liquid–solid interface under simple and mild conditions (Figure 5) [361]. They demonstrate the mild synthesis of five different highly crystalline two-dimensional COFs at room temperature using amino and aldehyde precursors with different geometric configurations. The resulting COF films have a large lateral size, controllable thickness, and high crystallinity. In addition, they are uniform and free of contamination, wrinkles, and damage when viewed under an optical microscope. Furthermore, they could be formed directly on the device substrate by interface engineering. The latter property is particularly advantageous for the fabrication of device arrays. For example, molecular dynamics simulations have shown that the presence of –OH groups on the solid surfaces strengthens the interaction energy between the substrate surface and the precursor. Promoting adsorption of precursors is advantageous for thin film fabrication. In other words, by selectively treating the substrate, pattern growth of COF films can be achieved. Furthermore, the COF films exhibit excellent chemical stability in DMF, MeOH, THF, H_2_O, NaOH, and HCl solutions and can withstand temperatures as high as 450 °C. COF films have high crystallinity and photoelectrochemical performance. The excellent stability is also beneficial for photoelectrochemical applications. 4,4′,4′,4′-(1,3,6,8-tetrakis(4-aminophenyl)pyrene and terephthalaldehyde. When used as active materials in electronic devices, they showed efficient responses. The obtained results are comparable to those of several metal-containing inorganic and carbon materials. When used as an active material in synaptic devices, excellent synaptic plasticity properties were obtained with light stimulation. Biological synaptic functions, such as short-term plasticity, long-term plasticity, and conversion from short-term to long-term plasticity, as well as superior synaptic plasticity properties induced by light stimulation, were successfully simulated. The liquid interfacial nanoarchitectonics method developed in this study will also lead to the large-scale facilitated preparation of COF membranes. It is expected to pave the way for multifunctional applications in optoelectronics, demonstrating their functionality.

**Figure 6:**
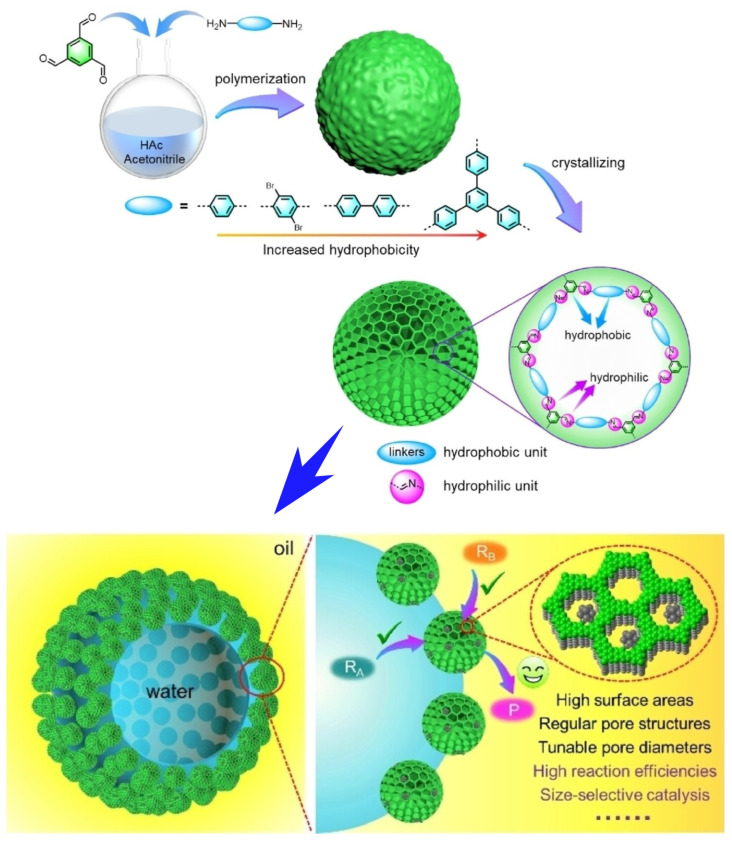
Preparation of COF structure on Pickering emulsion as controllable catalysts with a high surface area and tunable pore diameter to provide high reaction efficiency and excellent size selectivity. Structure fabrication method (**top**) and properties and functions (**bottom**) are explained. Reprinted with permission from [362]. Copyright 2024 Wiley-VCH.

An advanced reaction system of interest is the Pickering emulsion stabilized by solid particles. Such Pickering emulsions increase the oil–water interface and the liquid–liquid interface. As a result, they promote two-phase catalytic reactions. At the emulsion–liquid interface, the microenvironment and nanospatial properties of the reaction are controlled. Accordingly, reaction selectivity can be controlled. The compartmentalized droplet geometry can also be used to process continuous flow catalytic reactions. The use of advanced amphiphilic solid catalysts is essential for the development of superior Pickering emulsion catalysts. To form stable Pickering emulsions, substances adsorbing at the oil–water interface need to have appropriate amphiphilicity. Liquid interfacial nanoarchitectonics of such substances is desired. Zou, Fang, and co-workers showed that COF nanoparticles synthesized with highly hydrophobic monomers as linkers have excellent amphiphilic properties [362]. Furthermore, they showed that this COF structure is useful for Pickering emulsification catalysts (Figure 6). The amphiphilic COFs developed here can be used as solid emulsifiers to control emulsion type and droplet size. More importantly, the particle size of these COF nanoparticles is highly controllable. In other words, with high surface area and tunable pore diameter, catalytic systems with high reaction efficiency and excellent size selectivity can be achieved. For proof of concept, alcohol oxidation under two-phase conditions was investigated. For this purpose, Pd nanoparticles were encapsulated within these amphiphilic COF nanoparticles. The nanoarchitectonically prepared catalyst exhibited a catalytic efficiency that was 3.9 times higher than that of conventional amphiphilic solid catalysts. The significant increase in catalytic activity is still attributed to the high surface area and regular porous structure of the COFs. The nanoarchitectonics of the COFs is thought to be responsible for the assembly of fast channels that facilitate mass transfer at the Pickering droplet interface. The construction of COF structures by liquid interfacial nanoarchitectonics is expected to provide an innovative platform for Pickering emulsification catalysis.

**Figure 7:**
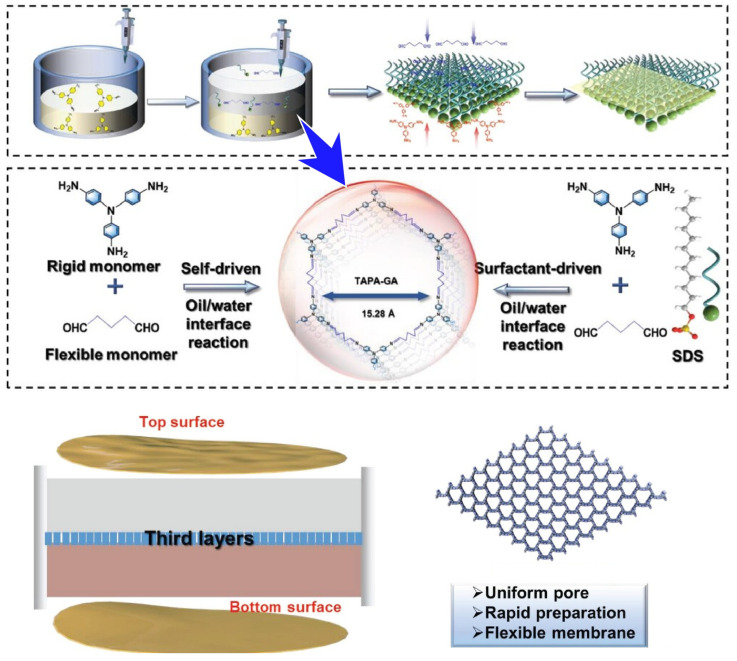
Synthesis of a two-dimensional soft COF (SCOF) based on an imine structure using a flex-ible linker of aldehydes and triangular building blocks, where sodium dodecyl sulfate molecules facilitate the migration of tris(4-aminophenyl)amine, making the contact between the amine and aldehyde monomers faster and more homogeneous. Reprinted with permission from [363]. Copyright 2023 Wiley-VCH.

Highly flexible and robust self-assembled COF membranes are used in a variety of applications. An example is their application as precision separation membranes. However, the synthesis of stable and functional COF membranes that actually exhibit useful functions is, surprisingly, technically challenging. Liu and co-workers synthesized a two-dimensional soft COF (SCOF) based on an imine structure using a flexible linker of aldehydes and triangular building blocks (Figure 7) [363]. This technique yields very large-area (two-dimensional and soft) COF molecular membranes. The synthesis uses a liquid–liquid interface consisting of water/dichloromethane as the reaction field. Rigid tris(4-aminophenyl)amine and flexible glutaraldehyde monomer are used as building blocks for the two-dimensional COF membrane. Sodium dodecyl sulfate is oriented at this liquid interface, and structures such as molecular crosslinks are self-assembled. The sodium dodecyl sulfate molecules facilitate the migration of tris(4-aminophenyl)amine, making the contact between the amine and aldehyde monomers faster and more homogeneous. This led to a record-breaking rate of preparation of the COF membranes and the appearance of more uniform pores. The nanoarchitectonically prepared two-dimensional COF membranes showed excellent sieving ability for small molecules. They were also resistant to strong alkalis (5 M NaOH), acids (0.1 M HCl), and various organic solutions. It also had the necessary flexibility as a membrane for practical use. The porous two-dimensional COF membranes are easy to fabricate and the pore size can be adjusted. As a result, they have the functional property of allowing precise molecular sieving. This nanoarchitectonics of the separation membrane was programmed from building blocks at the molecular level. The structures created have great potential for trace contaminant removal, drug/organic solution separation, oil/water separation, metal ion screening, alcohol/water separation, gas separation, and chiral discriminations.

## 4. Living Cell

Liquid interfaces can also contribute to a variety of biotechnology-related controls. One of the more complex systems is the control of cell culture and associated cell differentiation. In other words, nanoarchitectonics can be applied even at the living cell level of complexity. Cell culture is usually carried out at the interface between liquid and solid, such as the interface between solid surfaces and culture medium. The cells detect the mechanical properties of the solid interface, which in turn control their behavior and function [364,365,366]. This area of research is also being developed as mechanobiology [367,368,369]. Recently, pioneering systems that do not use solid interfaces have been reported. The interface between immiscible liquids is used as a culture environment for cells. In particular, organic solvents, such as perfluorocarbons, which do not adversely affect cells, are used as organic solvent layers [370]. It is now known that structures such as protein molecular membranes can be spontaneously nanoarchitectonized at such a liquid–liquid interface and influence cell differentiation [371]. Cells are influenced by the environment with which they come into contact to alter their behavior and function. It has been known that static properties such as the stiffness, nanotopography, and geometry of the materials with which cells come in contact have a significant impact. In addition, the viscoelastic properties of the matrix with stress relaxation are important in controlling cell fate and activity.

**Figure 8:**
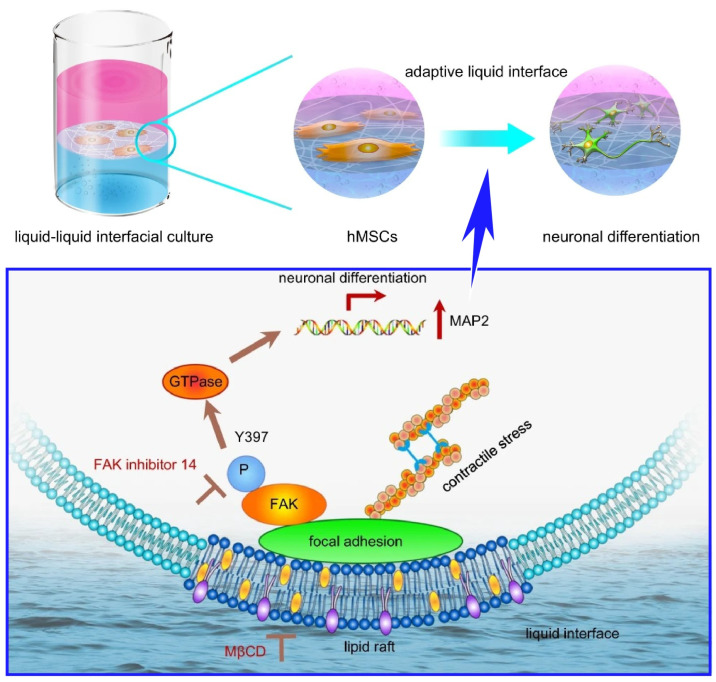
Effect of a two-dimensional network of protein nanofibrils spontaneously formed at the liquid–liquid interface composed of aqueous culture medium and fluorocarbons on stem cell behaviors, where lipid raft microdomains play a central role in both the initial cell adhesion and neural differentiation of stem cells through the integration of downstream signals involving focal adhesion kinase. Reproduced under terms of the CC-BY license [372]. Copyright 2022 Springer-Nature.

As an example, stem cells interact with the extracellular matrix and remodel it, which determines the fate of stem cells. The liquid–liquid interface, which is unaffected by the solid substrate, can be an ideal environmental site in which to explore the nature of cell behaviors. Jia et al. studied the effect of a two-dimensional network of protein nanofibrils spontaneously formed at the liquid–liquid interface composed of aqueous culture medium and fluorocarbons on stem cell behaviors (Figure 8) [372]. In particular, it was found that lipid raft formation and phosphorylation of focal adhesion kinase affect stem cell differentiation at the liquid–liquid interface. Protein nanofibrils are on the order of several microns in length and are in a form of polymeric β-sheet aggregates of proteins. They are biocompatible for cell adhesion and have high mechanical strength, which can mimic natural extracellular matrix fibers. Culturing stem cells on a two-dimensional network of protein nanofibrils at the liquid–liquid interface of water–perfluorocarbon promoted neural differentiation. In this process, lipid raft microdomains play a central role in both the initial cell adhesion and neural differentiation of stem cells. Lipid rafts help contact the cell membrane by containing cell adhesion molecules and provide a site for the enrichment of functional sites. They aid in the formation of large signaling complexes. As a result, stem cells are able to rapidly adapt to their constantly changing microenvironment. They integrate downstream signals involving focal adhesion kinase in lipid rafts within membrane microdomains. As a result, neurogenesis of stem cells is induced. The example shown here is just one example. The nanoarchitectonics of liquid interfaces incorporating bioactive proteins and responsive polymers will likely find applications in regenerative medicine and tissue engineering. Liquid–liquid interfaces can contribute to the development of nanoarchitectonics for previously unimagined adaptive biomaterials.

As illustrated in the examples above, cells also exhibit mechanosensing behavior at the liquid–liquid interface. Cell adhesion at the liquid–liquid interface is often mediated by the protein nanolayers derived from the culture medium that form at the interface. The media that form the liquid–liquid interface are not limited to the phase-separated structures of water and perfluorocarbons. Ueki et al. have developed a liquid–liquid interface culture technique using hydrophobic ionic liquids as the water-immiscible phase [373]. The diversity of ionic liquids is wide. By changing the chemical structure of the components or the combination of ions, their properties can vary greatly. The range of physicochemical parameters such as polarity, viscosity, surface tension, and ionic properties can be greatly varied. From this perspective, ionic liquids are often referred to as designer solvents. Their advantage over perfluorocarbons is their high solubility in a wide variety of substances. This allows for adaptation of cell control at the liquid–liquid interface. From among the diverse options, alkylphosphonium-type ionic liquids were found to be promising as ionic liquids with negligible cytotoxicity. The van der Waals interactions of the constituent ions and the charge distribution of the cations were investigated in terms of protein nanolayer formation and cell adhesion behavior mediating cell culture. Such studies could be applied to emulsion systems with a wide range of liquid–liquid interfaces. Emulsion with cell culture medium as the continuous phase and hydrophobic liquid as the dispersed phase could be a cell culture method that does not require plastic dishes. Cell resources can be recovered by using a filtration process that does not require trypsin enzyme treatment, facilitating full automation of the cell culture process.

**Figure 9:**
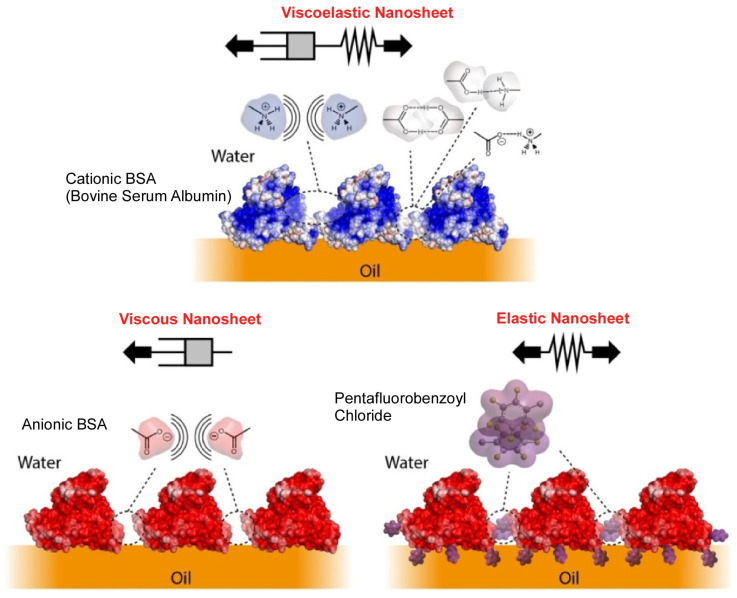
Protein nanosheets formed by co-assembling super-charged albumin with a pentafluorobenzoyl chloride surfactant, where coupling with pentafluorobenzoyl chloride surfactant results in strong interfacial elastic properties. Viscoelastic nanosheet (**top**), viscous nanosheet (**bottom left**), and elastic nanosheet (**bottom right**) are exemplified. Reproduced under terms of the CC-BY license [372]. Copyright 2023 American Chemical Society.

As a cell culture at the liquid–liquid interface, cell culture on micro-oil droplet surfaces is an environmentally benign alternative compared plastic dishes, which are implicated in the generation of microplastics. In addition, it is a strategy that can scale up the production of adherent cells by increasing the size of the system. The presence of proteins that function at the liquid interface is essential for cell culture on such micro-oil droplet surfaces. Gautrot and co-workers have developed a new class of protein nanosheets that serve these purposes by co-assembling supercharged albumin with a pentafluorobenzoyl chloride surfactant (Figure 9) [374]. It was found that the protein nanosheets formed by the liquid interfacial nanoarchitectonics mediate the adsorption of extracellular matrix proteins and cell adhesion. The behavior of the protein nanosheets was quantified by surface plasmon resonance and fluorescence microscopy. Supercharged albumin retains tension-active properties suitable for stabilizing microdroplets. Coupling with pentafluorobenzoyl chloride surfactant results in strong interfacial elastic properties. The protein nanosheet formed plays a dual role. It serves as a scaffold protein that structures the liquid–liquid interface and as a substrate for trapping extracellular matrix molecules. The adhesion and proliferation of human epidermal stem cells in bioemulsions stabilized by pinned droplets and supercharged nanosheets were also investigated. The methodology presented in this study results in a system that does not require plastics. It has the potential to revolutionize the cell manufacturing process and address environmental problems.

As discussed above, bioemulsions provide a promising venue for the growth of adherent cells in bioreactors. Their functionality depends on the development of protein nanosheets that are nanoarchitectonized at the liquid–liquid interface. Factors such as suitable interfacial mechanical properties and the promotion of integrin-mediated cell adhesion are important. Gautrot and co-workers investigated the effect of aliphatic surfactants such as palmitoyl chloride and sebacoyl chloride on the nanosheets formed by poly(l-lysine) at the liquid interface [375]. The assembly kinetics, interfacial shear dynamics, and viscoelasticity of poly(l-lysine) at the silicone oil interface were investigated. Using immunostaining and fluorescence microscopy, the effect of poly(l-lysine) nanosheets on mesenchymal stem cell adhesion was examined. The results revealed that the classical focal adhesion-actin cytoskeleton mechanism is involved. The proliferation of mesenchymal stem cells at the interface of other non-fluorinated oils, based on mineral and vegetable oils, was also investigated. Mesenchymal stem cells also developed focal adhesion at the silicone oil interface stabilized with poly(l-lysine) nanosheets. In doing so, they formed a mature actin cytoskeleton. Successful cell culture at non-fluorinated bioemulsion interfaces and systematic characterization of cellular phenotypes are also expected. Valuable insights were gained toward the realization of stem cell culture on mineral and plant-derived oil surfaces relevant to the healthcare and cultured meat industry. There are also many advantages from an engineering perspective. For example, simply centrifuging or filtering the cultures would facilitate the recovery of cellular products from the bioemulsion and subsequent processes.

## 5. Frontier Research, Organic Semiconductor

As the final example of liquid interface nanoarchitectonics, doping of organic semiconductors at the liquid–solid interface is described in this section. In semiconductor science and industry, inorganic semiconductors have taken the lead [376,377,378]. The development of the semiconductor industry is also an important part of national strategy. Therefore, technologies related to inorganic semiconductors have developed ahead of their time. On the other hand, it is said that technological development is approaching its limits. As an alternative, the development of organic semiconductor technology is desired [379,380,381]. The advantage of organic semiconductors is that they are easy to process, cheap, and can be mass produced. Another advantage is that flexible devices can be made. Electronic devices can be produced by low-cost printing such as inkjet printing. For this reason, their use in various flexible devices such as film sensors, electronic circuits, solar cells, light-emitting diodes, displays, and biological sensing devices are being studied worldwide. On the other hand, they are far inferior to inorganic semiconductors in terms of electrical conductivity and other properties. Therefore, the process of doping, which controls the ease of electricity flow in semiconductors, is essential for organic semiconductor materials [382,383,384]. For example, doping in organic semiconductors has been performed by chemical doping using reactions with redox reagents. However, redox reagents are easily degraded by reactions with water in the air. Therefore, chemical doping often requires special facilities to handle reagents in a vacuum or nitrogen atmosphere. In addition, problems have arisen regarding the accuracy and reproducibility of chemical doping due to the instability of redox reagents. These are major barriers to the fabrication of flexible devices and industrial applications using organic semiconductors. Innovative advances in doping technology for organic semiconductor thin films need to be made through liquid interfacial nanoarchitectonics.

**Figure 10:**
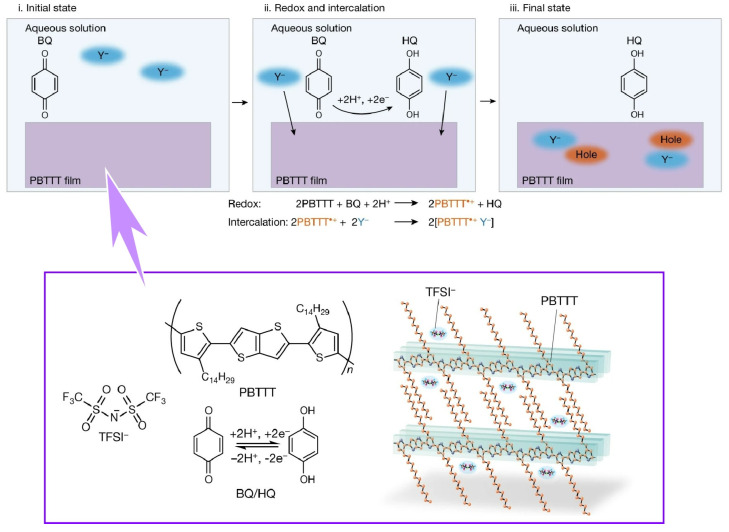
The synergistic reaction between proton coupling electron transfer reaction and ion intercalation leading to efficient chemical doping of crystalline organic semiconductor thin films under room temperature conditions. The doping mechanism (**top**) and chemical structure (**bottom**) are exemplified. Reprinted with permission from [385]. Copyright 2023 Springer-Nature.

With this background, Ishii, Yamashita, and co-workers developed a chemical doping process controlled by proton activity in water (Figure 10) [385]. Chemical doping is based on electron transfer reactions between molecular semiconductors and dopant molecules. In this case, the redox potential of the dopant is the key to controlling the Fermi level of the semiconductor. In this new method, doping conjugated with proton coupling electron transfer reaction is proposed. The synergistic reaction between proton coupling electron transfer reaction and ion intercalation has led to efficient chemical doping of crystalline organic semiconductor thin films under room temperature conditions. Polymeric organic semiconductor thin films were immersed in an aqueous solution containing a benzoquinone/hydroquinone system and hydrophobic molecular ions. When conversing from benzoquinone to hydroquinone, electrons are taken from the organic semiconductor, and holes are injected into the organic semiconductor at that time. At the same time, hole injection is stabilized by intercalation of hydrophobic ions. As the equilibrium between benzoquinone and hydroquinone is determined by the pH in the aqueous solution, the number of holes injected is precisely determined by the pH of the aqueous solution. The process is reproducible under normal temperature conditions in this pH-controlled aqueous solution. It shows unprecedented scalability, stability, and tunability. Chemical doping requires no special equipment for handling reagents in a vacuum or nitrogen atmosphere. This technology, which allows precise control of doping levels, is also expected to lead to the development of innovative sensors. Thin-film organic semiconductor sensors that measure pH and ion concentrations are expected to contribute to healthcare and biosensing technologies. Thus, the coupling of the nanoarchitectonics of liquid interfaces with chemical equilibrium and doping phenomena could bring innovation to the semiconductor industry.

## 6. Summary and Perspectives

In this paper, a brief description of nanoarchitectonics, which is a forefront concept in the development of artificial materials, is given. It then presented several recent examples that focused on the field of the liquid interface. Examples of the contributions of liquid interfacial nanoarchitectonics are as diverse as the examples given here. Examples include the creation of molecular complexes and assemblies, the construction of nano-regular structures such as MOFs and COFs, the control of living cells, and innovative doping techniques for organic semiconductors.

The diversity is due to the variety of elements that make up the liquid–liquid interface. In the case of liquid–liquid interfacial nanoarchitectonics, a variety of substances can be created that meet and form at the interface, depending on the components to be dissolved in each layer. Even simple molecules such as fullerenes can be nanoarchitectonically assembled in a variety of ways, depending on the choice of solvent and conditions. The resulting possibilities are endless. Until now, such approaches have been supported by simple mechanisms and the experience of researchers. Artificial intelligence (AI) will need to be introduced in order to make greater progress. Machine learning is being actively used to optimize the synthesis of materials and to elucidate the chemical phenomena that form the basis of such synthesis [386,387,388,389,390]. The need to integrate nanoarchitectonics and materials informatics is also being discussed [391,392]. Liquid interfacial nanoarchitectonics, with its diverse possibilities, is considered to be a very meaningful subject for the introduction of AI.

An additional possibility for the future of liquid interfacial nanoarchitectonics would be industrial applications. Many of the functions created by liquid interfacial nanoarchitectonics have potential applications ranging from biofunctions to device functions. Therefore, industrial applications should be considered as a goal. For this purpose, it is important to be able to mass produce at low cost. As seen in several examples, the use of emulsion technology with a wide range of liquid–liquid interfaces is key. Nanoarchitectonics at the interface of emulsions dispersed at the micro- or nanoscale, rather than at the visible interface created by two immiscible solvents in a sample bottle, will pave the way for simple mass production. Methodologies such as emulsion nanoarchitectonics, which is an extension of liquid interfacial nanoarchitectonics, should be further developed. If such technologies can be developed, we can expect to see the creation of technologies that contribute to industrial applications while taking advantage of the two major characteristics of liquids and interfaces, as seen in the organization of biological systems.

Finally, several concluding remarks on more general points for further developments of nanoarchitectonics approach are described. Two issues necessitate consideration. The first necessity is the establishment of nanoarchitectonics processes based upon strong theoretical backgrounds. For example, the formation of assembled structures with amphiphiles can be interpreted by theoretical descriptions [393]. In addition, evaluation and analyses of assembling motifs can be supported by theory [394,395,396]. These theoretical methodologies must be aggressively used in nanoarchitectonics approaches. Another necessity is the integration of nanoarchitectonics into current science and technology. The use of nanoarchitectonics as a new concept in currently existing molecular and materials systems would be an efficient way to accelerate nanoarchitectonics approaches. For example, the integration of molecular construction with metallacycle [397,398,399] into nanoarchitectonics would result in fruitful outputs. The coupling of the new nanoarchitectonics concept and current science and technology would be an effective strategy for many current problems facing the energy, environmental, and biomedical fields. These practically effective approaches could become emerging trends in science and technology.
